# Global Prevalence of Mitral Regurgitation: A Systematic Review and Meta-Analysis of Population-Based Studies

**DOI:** 10.3390/jcm14082749

**Published:** 2025-04-16

**Authors:** Gisella Figlioli, Alessandro Sticchi, Maria Nefeli Christodoulou, Andreas Hadjidemetriou, Gabriel Amorim Moreira Alves, Marco De Carlo, Fabien Praz, Raffaele De Caterina, Georgios K. Nikolopoulos, Stefanos Bonovas, Daniele Piovani

**Affiliations:** 1 Department of Biomedical Sciences, Humanitas University, Pieve Emanuele, 20072 Milan, Italy; 2 IRCCS Humanitas Research Hospital, Rozzano, 20089 Milan, Italy; 3 Pisa University Hospital, University of Pisa, 56126 Pisa, Italy; 4 West Suffolk NHS Foundation Trust, Bury Saint Edmunds IP33 2QZ, UK; 5 Mersey and West Lancashire Teaching Hospitals NHS Trust, Southport L35 5DR, UK; 6 Department of Cardiology, Bern University Hospital, University of Bern, 3012 Bern, Switzerland; 7 Medical School, University of Cyprus, Nicosia 1678, Cyprus

**Keywords:** mitral valve insufficiency, heart valve diseases, epidemiology, cross-sectional studies

## Abstract

**Background/Objectives**: Mitral regurgitation (MR) is the most common left heart valve disease, but its exact prevalence remains uncertain. To estimate the prevalence of MR we conducted a systematic review and meta-analysis of population-based studies. **Methods**: We searched the Medline/PubMed, Embase, and Scopus databases, in January 2023, for studies reporting or allowing for the calculation of the prevalence of moderate-to-severe MR in the general population. Eligible studies included those using echocardiography or primary care databases from countries with universal healthcare. Studies where echocardiography was performed for medical indications were excluded. Random-effects meta-analysis was used to calculate the pooled estimates. Subgroup and meta-regression analyses were employed to investigate the reasons for heterogeneity. Mixed-model multivariable meta-regression was used to estimate age- and sex-specific prevalence. **Results**: After screening 13,847 records, we identified 20 eligible studies (22 study populations) including 6,036,691 individuals. The global prevalence of moderate-to-severe MR was 0.67% (95% CI, 0.33−1.11). Prevalence increased greatly with age, and it was estimated to be approximately 0.63% (0.25–1.16) at age 50, 2.85% (1.96–3.90) at 70, and 6.45% (4.17–9.16) by 90 years. North America showed the largest crude prevalence (1.11%; 0.52−1.88), followed by Europe (0.60%; 0.34−0.92), Asia (0.24%; 0.00−0.92), and Africa (0.16%; 0.03−0.37). Differences in prevalence by geographic region and ethnic group were primarily attributable to population age. Prevalence did not differ by sex, study year, or diagnostic criteria. **Conclusions**: Moderate-to-severe MR is a prevalent condition, particularly among elderly people. With rising life expectancy worldwide, ensuring universal access to interventions will be vital to reduce morbidity and mortality.

## 1. Introduction

Mitral regurgitation (MR), the backward blood flow from the left ventricle into the left atrium during systole, is the most common left heart valve disease worldwide [1,2]. While mild MR is often seen in healthy individuals, moderate-to-severe MR affects an estimated 2.5 million people in the United States [3]. Clinically significant MR is associated with a high risk of heart failure following diagnosis and markedly increased morbidity and mortality [1].

The main therapeutic options for MR, when it reaches a degree of severity, are surgical, either through mitral valve replacement or repair [4]. Advances in surgical techniques, risk stratification, and patient outcome tracking have greatly improved prognosis when timely diagnosis occurs, allowing many patients to reach life expectancies similar to those without the condition [5]. However, MR remains underdiagnosed and undertreated, resulting in poor survival outcomes for many patients [6]. As population aging continues, MR prevalence is expected to rise, underscoring its importance as a major public health concern [3]. This challenge is compounded by the lack of comprehensive, population-based data on the global burden of MR. Most available epidemiologic studies focus primarily on Western countries and often rely on older data [3,7,8,9]. To effectively allocate resources and guide healthcare investments in specialized diagnostic and treatment services for patients with MR, clinicians and policy makers require updated evidence regarding the burden of moderate-to-severe MR in the general population. Such data are essential for informed decision-making about health system spending and prioritizing interventions, including strategies to improve general awareness around the condition.

We conducted a systematic review and meta-analysis of population-based studies to assess the global prevalence of moderate-to-severe MR in the general adult population with the aim of quantifying the global burden of this condition by age, sex, and across geographic areas.

## 2. Methods

This work is reported according to the Preferred Reporting Items for Systematic Reviews and Meta-Analyses (PRISMA) guideline [10]. The protocol was registered with PROSPERO (CRD42023448216).

### 2.1. Search Strategy

We systematically searched for population-based studies reporting the prevalence of MR in the Medline/PubMed, Embase, and Scopus databases from inception to 17 January 2023. Key search terms included “mitral regurgitation” OR “mitral disease” OR “mitral insufficiency” OR (“mitral valve” AND “regurgitation”) combined with “prevalence” OR “incidence” OR “epidemiology” OR “surveillance” OR “survey”. We used the appropriate MeSH terms, truncated terms, variants, and acronyms. The search algorithms are reported in Appendix S1. We excluded editorials, congress abstracts, and studies not conducted in humans. We screened the reference lists of relevant systematic or narrative reviews to identify additional articles. We also consulted with field experts to gather additional evidence. No language restrictions were imposed.

### 2.2. Selection Criteria

We considered as eligible cross-sectional or longitudinal studies collecting data with a well-specified sampling frame, including population samples of adults at the national, subnational, or community level of reporting, or allowing for the calculation of the prevalence of moderate-to-severe MR. Studies using Doppler echocardiography for diagnosis were considered the most appropriate to answer our research question. Given the broad temporal span of the included studies, we considered studies with slight variations in diagnostic criteria for the identification of our target condition (Supplementary Table S1). A brief discussion of the temporal evolution of the definition and assessment methods of moderate-to-severe MR is given in Appendix S2 [11,12,13,14,15,16].

Also, we considered eligible studies using primary care databases conducted in countries with universal healthcare coverage or countries where at least 80% of the population was publicly insured. To be considered eligible, such studies should have used the International Classification of Diseases (ICD) or other well-defined classification systems, allowing the accurate identification of MR.

We excluded studies that did not include a representative sample of the general adult population, particularly those with an increased or reduced likelihood of presenting with MR. More in detail, echocardiographic studies were excluded if participants were selected based on symptoms, suspected valvular diseases, or any medical indication suggesting the need for diagnostic evaluation. Studies conducted explicitly on elderly people were still retained if diagnostic procedures or study inclusion were not prompted by symptoms or established medical conditions. These studies were excluded from the primary analysis to avoid introducing heterogeneity that could inflate uncertainty in our global estimate, as they represented a distinct subgroup with higher expected prevalence. Nevertheless, these were retained for meta-regression analysis to inform age- and sex-specific prevalence estimates. Studies focusing on individuals <18 years old were considered ineligible.

Four authors (G.F., A.S., M.N.C., and A.H.) independently screened the titles and abstracts, excluding irrelevant studies. Articles identified as potentially eligible were read in full and critically appraised for inclusion. Disagreements were resolved by consensus with the assistance of the senior authors (S.B. and D.P.).

### 2.3. Data Extraction

Two authors (G.F. and A.S.) independently extracted data using a predefined extraction form. The following data were extracted from each study: first author name, publication title and year, study period, study design, country, predominant ethnicity, method/criteria used for identifying MR, mean age and age range, proportion of males, number of participants, and number of cases with MR. Any stratified data by age, sex, or ethnic group was also extracted from the articles’ main texts and supplementary materials. If the mean study age was not specified, we derived it from the age-stratified data. If ethnicity was not specified, we assessed whether the study population likely reflected the country’s demographics and assumed that participants belonged predominantly to the most represented country’s ethnic group.

### 2.4. Quality Assessment

A quality assessment of the included studies was performed independently in duplicate, using a modified version of the Joanna Briggs Institute Prevalence Critical Appraisal Tool [17].

### 2.5. Statistical Analysis

Confidence intervals (CI) for prevalence in each individual study were calculated using the exact binomial method (Clopper–Pearson). The double arcsine transformation was applied prior to data synthesis to prevent variance instability [18]. This transformation does not require continuity correction in the case of studies with zero cases. To allow interpretability, the pooled prevalence was back-transformed on the normal scale using the inverse Freeman–Tukey transformation [19].

The pooled prevalence was estimated by random-effects meta-analysis. The random-effects model was chosen over the fixed-effects model as it accounts for both chance variations and true differences in prevalence across studies. Studies were weighted using the inverse variance method. The restricted maximum-likelihood method was used for estimating τ-squared. Statistical heterogeneity across studies was assessed with the Cochran’s Q-test [20], with a conservative 0.10 level of significance. Heterogeneity was further assessed using the I-squared statistic. This was interpreted as follows: 0–40%, not important; 30–60%, moderate; 50–90%, substantial; and 75–100%, considerable heterogeneity [21]. The small-study effect was assessed visually with a funnel plot and using the Egger regression asymmetry test.

Subgroup and meta-regression analyses were conducted to explore potential sources of heterogeneity. Subgroup meta-analyses were performed by geographic area (i.e., continent), ethnicity of participants, method/criteria used for identifying MR, and study size. The subgroup analysis by identification criteria was used to establish the appropriateness of pooling echocardiographic studies with large registry-based databases with a differential diagnostic standard. We assessed whether subgroups were associated with significantly different estimates using a test of interaction [22].

Univariable and multivariable mixed-effects meta-regression analyses were performed for the relevant continuous moderators as follows: mean age, proportion of males, and study year (midpoint of the study period). Bubble plots illustrated the independent associations between each moderator and the prevalence of moderate-to-severe MR, with study-specific effect sizes on the *y*-axis and moderator values on the *x*-axis. Marker sizes reflected study precision, and each graph included the (marginal) multivariable meta-regression lines with 95% CIs. To allow interpretability, graphs were plotted on the normal scale after applying the inverse Freeman–Tukey double arcsine transformation, using the harmonic mean of sample sizes [19].

We also obtained the (marginal) estimates for the moderate-to-severe prevalence of MR at crescent values of age from the multivariable meta-regression model considering the proportion of males and the study publication year kept at the weighted means. Similarly, we derived sex- and sex-and-age-specific estimates. Analyses and data visualization were performed using the R statistical software version 4.4.2, packages meta and metafor (R Core Team, https://www.r-project.org/). For all tests, except for heterogeneity, we considered a 2-tailed *p* < 0.05 as statistically significant.

## 3. Results

**Figure 1:**
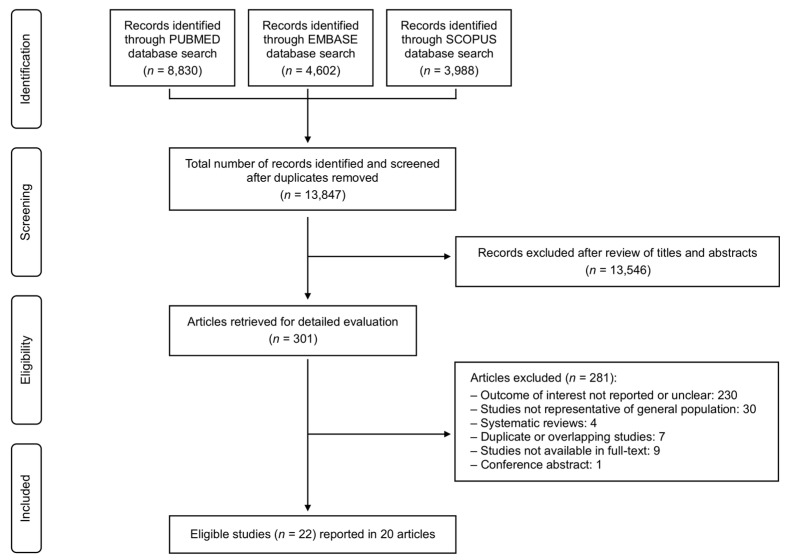
Summary of the evidence search and selection process (flowchart).

The systematic search identified 13,847 unique records. After screening the abstracts and reviewing the full texts, 20 studies covering 22 study populations were deemed eligible for inclusion (Figure 1) [3,8,9,23,24,25,26,27,28,29,30,31,32,33,34,35,36,37,38,39]. The primary reason for exclusion was the absence or unclear definition of moderate-to-severe MR prevalence data (N = 230), followed by the lack of representation of the general population, such as echocardiography studies where the diagnostic assessment was clearly medically indicated (N = 30).

The included studies comprised a combined sample of 6,036,691 individuals (median 2369; IQR 455–4728). Study characteristics are presented in Table 1, with a more detailed description of the method/criteria used for identifying MR provided in Supplementary Table S1. Quality assessment for each study is reported in Supplementary Table S2. Publication years ranged from 1989 to 2021, while study years ranged from 1988 to 2017. On average, there was a 6.7-year (95% CI, 4.7–8.6) delay between the period covered by each study and its publication. Most studies contributed data from North America (eight studies, nine study populations) [3,23−27,34,35] and Europe (eight studies) [8,9,28−32,37], followed by Asia (four studies) [30,33,36,39] and Africa (one study) [38]. The United States (eight studies, nine study populations) [3,23−27,34,35] and the United Kingdom (UK, four studies) [8,9,32,37] were the most frequently represented countries. Eighteen studies determined the diagnosis of MR using Doppler echocardiography [3,8,23−36,38,39], while two comprehensive record-linkage studies conducted in the UK identified cases through the relevant diagnostic codes [9,37]. The weighted mean age across all study populations was 44.9 years, with mean age per study ranging from 20 to 90 years old. Among the 5,916,298 individuals from six study populations that reported sex-specific data, 2,662,092 (45.0%) were females. All studies either reported or allowed for the derivation of the proportion of males.

### Data Synthesis

Five studies focused explicitly on elderly people and showed the mean age of participants above 70 years [8,29,31,32,39]; therefore, our analysis for the general adult population was conducted on 15 studies including 17 study populations (N = 6,029,659) [3,9,23−28,30,33−38].

**Figure 2:**
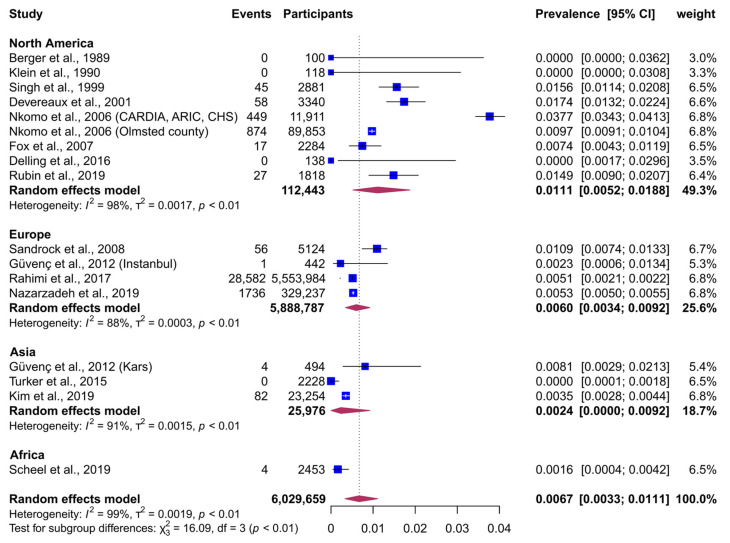
Random-effects meta-analysis of the prevalence of moderate-to-severe mitral regurgitation, grouped by geographic continent [3,9,23,24,25,26,27,28,30,33,34,35,36,37,38]. Abbreviation: CI, Confidence Interval.

The pooled global prevalence of moderate-to-severe MR was 0.67% (95% CI, 0.33−1.11; Figure 2). We noted considerable heterogeneity in this analysis (τ^2^ = 0.002; I^2^ = 98.6%; *p* < 0.001). No evidence of small-study effect highlighted by funnel plot asymmetry was observed (Supplementary Figure S1; P_Egger_ = 0.14). We investigated reasons for heterogeneity in subgroup and meta-regression analyses (Table 2 and Table 3).

Subgroup analyses suggested a significant geographic disparity in the prevalence of moderate-to-severe MR (Figure 2, Table 2, *p* = 0.001). North America showed the largest prevalence (1.11%, 95% CI, 0.52−1.88), followed by Europe (0.60%; 0.34−0.92), Asia (0.24%; 0.00−0.92), and Africa (0.16%; 0.03−0.37). Similarly, prevalence differed significantly by ethnic group (Table 2, Supplementary Figure S2, *p* < 0.001), with the only study conducted on American Indian individuals showing the largest estimates [26], followed by the Hispanic [35], White [3,8,9,23−25,28−34,37], Black [27,38], and Asian ethnic groups [36,39]. When grouped by method/criteria used for identifying MR, there was no significant difference in prevalence (*p* = 0.18) between studies using Doppler echocardiography (0.69%, 95% CI, 0.31–1.21) [3,8,23−36,38,39] and record-linkage studies using ICD-10 codes and having confirmed most cases by echocardiography (0.52%, 95% CI, 0.51–0.52) [9,37]. Additionally, no significant difference was observed by study size.

Meta-regression analyses comprised all studies, including those performed on elderly populations, and considered age- and sex-stratified estimates whenever available. Age was the only significant moderator of heterogeneity across studies (Table 3; *p* < 0.001). Although age alone explained a large proportion of the variability in prevalence across studies (R-squared = 47.2%), there was still a significant amount of residual heterogeneity (τ^2^ = 0.005, SE = 0.001; χ^2^ = 4976; *p* < 0.001). The analyses did not show any significant temporal trend or statistically significant difference by sex in the prevalence of moderate-to-severe MR (Table 3).

Based on the results of multivariable meta-regression analysis, the estimated global prevalence of moderate-to-severe MR was approximately 0.08% (95% CI, 0.00–0.50) at 40 years of age, increasing to 0.63% (95% CI, 0.25–1.16) at age 50, 1.56% (95% CI, 1.01–2.22) at 60, 2.85% (95% CI, 1.96–3.90) at 70, 4.48% (95% CI, 3.00–6.23) at 80, and reaching 6.45% (95% CI, 4.17–9.16) by 90 years. The estimated global prevalence by sex was 0.55% (95% CI, 0.05–1.41) in females and 0.63% (95% CI, 0.06–1.61) in males. Although sex did not significantly alter the estimates, we present age-specific, sex-specific, and age-and-sex-specific prevalence estimates in Table 4 for illustrative purposes.

**Figure 3:**
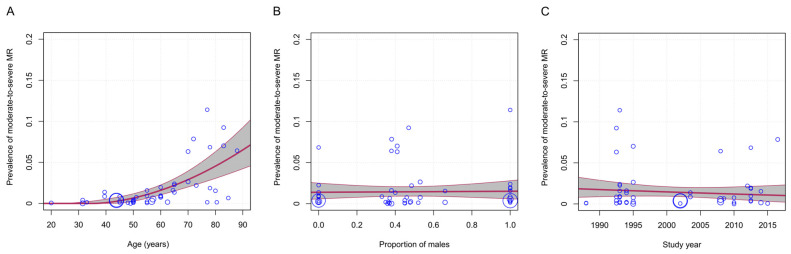
Bubble plots from multivariable meta-regression analysis, illustrating the independent associations of the mean age (**A**), proportion of males (**B**), and study year (**C**), with the prevalence of moderate-to-severe mitral regurgitation. The study-specific effect size is displayed on the y-axis and the moderator of interest on the x-axis. The size of the markers is proportional to the size of each study. The graphs are accompanied by the (marginal) multivariable meta-regression lines in red and the respective 95% confidence intervals in gray. The bubble representing the study by van Bemmel et al. (2010) [29] is omitted to enhance visual clarity, though its data were fully incorporated into the analysis.

Figure 3 presents bubble plots showing the adjusted association of age, proportion of males, and study year with the prevalence of moderate-to-severe MR. The meta-regression lines in these graphs depict the “marginal” relationship between each continuous predictor and prevalence, holding all other moderators constant at their weighted mean values. Bubble plots of the univariable meta-regression results for each continuous moderator are shown in Supplementary Figures S3−S5.

Sensitivity meta-regression analyses, incorporating either the study continent or participant ethnicity into the multivariable model, showed no significant deviation from the main model’s findings (see Supplementary Tables S3 and S4).

## 4. Discussion

This systematic review and meta-analysis of population-based studies estimated the global prevalence of moderate-to-severe MR in the general adult population. Age was identified as the primary factor influencing MR prevalence across societies. While crude prevalence varied by geographic region and ethnicity, these differences were largely attributable to age distribution. This is the first meta-analysis to present global, population-based, age- and sex-specific estimates of clinically significant MR.

Our findings should be compared with those of Yadgir et al., who used Bayesian modeling in the Global Burden of Disease Study 2017 [40]. They estimated the prevalence of degenerative mitral valve diseases at approximately 0.2%, slightly lower but within the lower bound of our confidence interval. Unlike our analysis, which primarily relied on echocardiographic studies conducted in non-medically indicated general populations, Yadgir et al. utilized diverse direct and indirect data sources, with echocardiographic studies forming a minority [40]. In regions with limited healthcare access, such as non-Western settings, reliance on administrative data, clinical records, and other indirect data sources may underreport subclinical cases. Conversely, population-based echocardiographic studies are better at capturing the true burden of disease, identifying cases that might otherwise go undiagnosed, even in developing countries. Detection bias, particularly due to disparities in healthcare access, might explain the modest difference between our findings and those of Yadgir et al. [40].

We observed geographic differences, with higher prevalence estimates in North America and Europe compared to Asia and Africa, along with significant variations by ethnicity. However, these disparities were no longer significant after accounting for age. This contrasts with Yadgir et al. [40], where estimates differed across regions with varying levels of development, even after age standardization. We believe that both sets of results are valuable but require different interpretations. Yadgir’s results, influenced by diverse data sources and healthcare access disparities, likely reflect the “visible” burden of disease. In contrast, our estimates aim to approximate the “true” burden, including undiagnosed cases in resource-limited settings, despite the inherent uncertainties.

We found no significant sex differences in MR prevalence, consistent with the complex interplay of sex-specific risk factors for primary and secondary MR [41]. Primary MR tends to affect females more [42], while secondary MR is likely more common in males due to increased smoking and alcohol use [43,44]. Conversely, obesity—a key factor for secondary MR—is more prevalent among females [45], and hypertension rates are similar between sexes [46]. Although there are other biological factors specific to each sex [41], the balance across modifiable risk factors likely accounts for a similar global MR prevalence observed between males and females. This aligns with the findings of Yousuf et al., who attributed the higher crude MR prevalence in females to their longer life expectancy [40]. However, regional disparities in risk factor distribution and genetic predisposition to MR [47] may still contribute to sex-based differences in certain populations.

This study has several strengths. We pre-registered our protocol and conducted a robust systematic search across three major scientific databases. Four investigators independently performed the search, with data extractions completed in duplicate. By including studies with a wide age range, we enhanced the generalizability of our findings to the general adult population. Notably, this is the first meta-analysis to report population-based, age- and sex-specific global prevalence estimates for moderate-to-severe MR.

We primarily included studies using Doppler echocardiography, the gold standard for diagnosing MR, along with two high-quality record-linkage studies from the UK that identified cases using relevant diagnostic codes. These studies were included under the assumption that all clinically significant MR cases would be captured in a high-income country with universal healthcare like the UK. Although not all cases in these studies were directly confirmed by Doppler echocardiography, an analysis restricted solely to echocardiographic studies yielded prevalence estimates closely aligned with our main results.

We carefully excluded studies where imaging or inclusion was based on medical indications, such as symptoms or concurrent conditions, to minimize selection bias that could inflate prevalence estimates. However, this strict exclusion resulted in a smaller pool of eligible studies, reducing statistical power for most subgroup analyses and limiting geographic coverage. Although data from four continents were included, the 22 study populations covered only nine countries, with limited representation from Asia and Africa and no data from South America and Oceania. More specifically, the majority of the studies were conducted in North America and Europe, representing approximately 6 million people, whereas the rest of the global population is represented by only 28,000 individuals. The Asian studies were confined to Korea and Turkey, and the one African study was conducted in Uganda; in both analyses these countries cannot be considered representative of their respective continents. As a result, these findings did not allow for reliable estimates of MR prevalence in Asia or Africa. This gap highlights the urgent need for additional, well-designed research in these regions.

Another limitation was the substantial statistical heterogeneity, leading to large confidence intervals and imprecise prevalence estimates, which is a common issue in the meta-analyses of prevalence studies [48,49,50]. While age explained about half of the heterogeneity, a significant portion remained unexplained. Variations in diagnostic tools, imaging techniques, and disease severity definitions—reflecting healthcare development differences across regions and time periods—may have contributed to this. Some heterogeneity may also be due to genuine differences in prevalence, potentially linked to unmeasured genetic or environmental factors specific to certain populations. Additionally, many primary studies had a significant delay between data collection and publication, and while our search was comprehensive, we could not identify more recent prevalence data. This limitation reflects gaps in primary data availability rather than flaws in the current synthesis.

## 5. Conclusions

Our study found that clinically significant MR is common, particularly among elderly people, with a non-linear association between age and prevalence. The scarcity of recent data, especially from certain regions, underscores the need for updated, high-quality epidemiological studies to better capture the global burden of MR. Advances in diagnostic techniques and increasing life expectancy make recent data crucial for assessing trends and potential changes in prevalence. This gap highlights the possibility that current data may underestimate MR prevalence in certain areas or populations. With the strong link between MR and age, countries experiencing rising life expectancy are likely to face an increasing MR burden. Preparing healthcare systems for the growing demand for diagnosis and treatment and improving universal access to interventions will be key to reducing morbidity and mortality, particularly in aging populations.

## Figures and Tables

**Table 1:** Study characteristics for the study populations included (N = 22).

Study	Country	Study Period (yrs)	Sample Size (N.)	Mean Age of Participants (yrs)	Males (%)	Method/Criteria for Identifying MR
Berger et al., 1989 [23]	USA	NR	100	45	52	Echocardiography
Klein et al., 1990 [24]	USA	1987−1988	118	48	44.9	Echocardiography
Singh et al., 1999 [25]	USA	1991−1995	2881	54	46.4	Echocardiography
Devereux et al., 2001 [26]	USA	1993−1995	3340	64.5	38	Echocardiography
Nkomo et al., 2006 [3]	USA	CARDIA: 1985−1992; ARIC: 1987−1995; CHS: 1989−1992	11,911	55.6	42.3	Echocardiography
Nkomo et al., 2006 [3]	USA (Olmsted County)	1990−2000	89,853	44.8	45.5	Echocardiography
Fox et al., 2007 [27]	USA	1993−1995	2284	60.0	38.2	Echocardiography
Sandrock et al., 2008 [28]	Germany	2001−2006	5124	39.5	78.9	Echocardiography
van Bemmel et al., 2010 [29]	The Netherlands	1997−1999	81	90	33	Echocardiography
Güvenç et al., 2012 [30]	Turkey (Istanbul, Europe)	NR	442	50	35	Echocardiography
Güvenç et al., 2012 [30]	Turkey (Kars, Asia)	NR	494	51.1	46	Echocardiography
Vaes et al., 2012 [31]	Belgium	2008−2009	556	84.7	37.1	Echocardiography
Yousaf et al., 2012 [32]	United Kingdom	2007−2009	357	87.9	38	Echocardiography
Turker et al., 2015 [33]	Turkey	2010	2228	49	36	Echocardiography
d’Arcy et al., 2016 [8]	United Kingdom	2012	2500	73	48.5	Echocardiography
Delling et al., 2016 [34]	USA	1996−2008	138	55	37	Echocardiography
Rahimi et al., 2017 [9]	United Kingdom	1990−2015	5,553,984	43.8	45.3	ICD-10, with most cases being confirmed by echocardiography
Rubin et al., 2019 [35]	USA	2011−2014	1818	55.2	42.3	Echocardiography
Kim et al., 2019 [36]	South Korea	2012−2016	23,254	63.3	66	Echocardiography
Nazarzadeh et al., 2019 [37]	United Kingdom	2006−2010	329,237	56.9	46	ICD-10 and UK biobank codes, with most cases being confirmed by echocardiography
Scheel et al., 2019 [38]	Uganda	NR	2453	20	45	Echocardiography
He et al., 2021 [39]	China	2015−2018	3538	72	38.1	Echocardiography

Abbreviations: ARIC, Atherosclerosis Risk in Communities (study); CHS, Cardiovascular Health Study; CARDIA, Coronary Artery Risk Development in Young Adults (study); NR, Not Reported; ICD, International Classification of Diseases.

**Table 2:** Subgroup meta-analysis of the prevalence of moderate-to-severe mitral regurgitation in adults by the pertinent categorical moderators (N = 17). ^1.^

Variable	N. of Studies	N. of Participants	Prevalence, % (95% CI)	*p*-Value ^2^
Continent				
Africa	1	2453	0.16 (0.03−0.37)	0.001
Asia	3	25,976	0.24 (0.00−0.92)	…
Europe	4	5,888,787	0.60 (0.34−0.92)	…
North America	9	112,443	1.11 (0.52−1.88)	…
Predominant Ethnicity				
American Indian	1	3340	1.74 (1.34−2.25)	<0.001
Asian	1	23,254	0.35 (0.26−0.42)	…
Black	2	4737	0.40 (0.03−1.16)	…
Hispanic	1	1818	1.49 (1.01−2.18)	…
White	12	5,996,510	0.62 (0.20−1.21)	…
Method/Criteria for Identifying Mitral Regurgitation				0.176
Doppler Echocardiography	15	146.438	0.69 (0.31–1.21)	…
ICD-10	2	5,883,221	0.52 (0.51–0.52)	…
Study Size				0.228
<1000	5	1292	0.22 (0.00–0.65)	…
≥1000	12	6,028,367	0.86 (0.39–1.50)	…

Footnotes: ^1^ Five studies conducted explicitly on elderly individuals were not included in this specific analysis. ^2^ Test of interaction. Abbreviation: CI, Confidence Interval.

**Table 3:** Univariable and multivariable mixed-effects meta-regression analysis investigating the association of mean age, proportion of males, and study year, with prevalence of moderate-to-severe mitral regurgitation.

	Univariable Meta-Regression Analysis				Multivariable Meta-Regression Analysis			
Variable	N. of Estimates	β Coefficient	(95% CI)	*p*-Value	N. of Estimates	β Coefficient	(95% CI)	*p*-Value
Age	58	+0.0038	(+0.0026, +0.0050)	<0.0001	50	+0.0043	(+0.0029, +0.0057)	<0.0001
Proportion of males	50	+0.0023	(−0.0895, +0.0941)	0.9605	50	+0.0049	(−0.0636, +0.0733)	0.8890
Study year	58	−0.0004	(−0.0036, +0.0028)	0.8189	50	−0.0011	(−0.0037, +0.0015)	0.4201

Footnote: Beta coefficients are presented on the Freeman–Tukey scale, which limits direct interpretability. Abbreviation: CI, Confidence Interval.

**Table 4:** Age- and sex-specific estimates of the global prevalence of moderate-to-severe mitral regurgitation. ^1^

	Females		Males		Overall	
Age	Predicted Prevalence, %	(95% CI)	Predicted Prevalence, %	(95% CI)	Predicted Prevalence, %	(95% CI)
30	0.00	(0.00−0.19)	0.00	(0.00−0.27)	0.00	(0.00−0.11)
40	0.06	(0.00−0.67)	0.10	(0.00−0.82)	0.08	(0.00−0.50)
50	0.59	(0.07−1.46)	0.68	(0.09−1.68)	0.63	(0.25−1.16)
60	1.50	(0.65−2.66)	1.63	(0.67−2.95)	1.56	(1.01−2.22)
70	2.77	(1.50−4.37)	2.93	(1.54−4.73)	2.85	(1.96−3.90)
80	4.38	(2.54−6.67)	4.58	(2.59−7.09)	4.48	(3.00−6.23)
90	6.32	(3.71−9.55)	6.57	(3.79−10.0)	6.45	(4.17−9.16)

Footnote: ^1^ Marginal prediction derived from multivariable meta-regression analysis by average age of participants, proportion of males in the study, and study year.

## Data Availability

Data available upon request from the authors.

## Supplementary Materials

The following supporting information can be downloaded at: https://www.mdpi.com/article/10.3390/jcm14082749/s1, Appendix S1. Search algorithms; Appendix S2. Temporal evolution of the definition and assessment methods of moderate-to-severe MR; Supplementary Table S1. Methods and criteria used for identifying mitral regurgitation; Supplementary Table S2. Quality assessment of the included studies; Supplementary Table S3. Sensitivity multivariable mixed-effects meta-regression analysis reporting the independent association of mean age, proportion of males, study year, and continent with the prevalence of moderate-to-severe mitral regurgitation; Supplementary Table S4. Sensitivity multivariable mixed-effects meta-regression analysis reporting the independent association of mean age, proportion of males, study year, and predominant ethnicity with the prevalence of moderate-to-severe mitral regurgitation; Supplementary Figure S1. Funnel plot of the prevalence of moderate-to-severe mitral regurgitation in adults (N = 17). Supplementary Figure S2. Subgroup analysis of the prevalence of moderate-to-severe mitral regurgitation in adults by the predominant ethnicity of the participants (N = 17); Supplementary Figure S3. Bubble plot from univariable meta-regression analysis, illustrating the association of the mean age with the prevalence of moderate-to-severe mitral regurgitation; Supplementary Figure S4. Bubble plot from univariable meta-regression analysis, illustrating the association of proportion of males with the prevalence of moderate-to-severe mitral regurgitation; Supplementary Figure S5. Bubble plot from the univariable meta-regression analysis, illustrating the association of study year with the prevalence of moderate-to-severe mitral regurgitation.

## Abbreviations

The following abbreviations are used in this manuscript:

CI	Confidence Interval
ICD	International Classification of Diseases
MR	Mitral Regurgitation
PRISMA	Preferred Reporting Items for Systematic Reviews and Meta-Analyses
UK	United Kingdom
