# Supplementary material for: Global Prevalence of Mitral Regurgitation: A Systematic Review and Meta-Analysis of Population-Based Studies
Source: J Clin Med. 2025 Apr 16;14(8):2749. doi: 10.3390/jcm14082749 (PMC12028080; doi:10.3390/jcm14082749)
Supplement: Supplementary file 1 [file jcm-14-02749-s001.zip › jcm-3577195-supplementary.pdf]

Appendix S1. Search algorithms.

PubMed/Medline

("Mitral Valve Insufficiency"[mesh] OR "mitral insufficiency"[tiab] OR "mitral valve insufficiency"[tiab] OR "mitral regurgitation"[tiab] OR "mitral valve regurgitation"[tiab] OR "mitral disease"[tiab] OR "mitral valve disease"[tiab] OR "mitral incompetence"[tiab] OR "mitral valve incompetence"[tiab] OR "Heart Valve Diseases/epidemiology"[MAJR]) AND (prevalence[mesh] OR prevalence[tiab] OR incidence[mesh] OR incidence[tiab] OR Epidemiology[sh] OR survey[tiab] OR population-based[tiab]) NOT (editorial[pt] OR congress[pt])
Filter: humans

Embase

('mitral valve regurgitation':ti,ab,kw OR 'mitral insufficiency':ti,ab OR 'mitral valve insufficiency':ti,ab OR 'mitral regurgitation':ti,ab OR 'mitral valve disease':ti,ab,kw OR 'mitral disease':ti,ab OR 'mitral incompetence':ti,ab OR 'mitral valve incompetence':ti,ab OR 'valvular heart disease'/dm_ep) AND ('prevalence':ti,ab,kw OR 'incidence':ti,ab,kw OR 'epidemiology'/mj OR 'survey':ti,ab OR 'population-based':ti,ab) NOT ('conference paper'/it OR 'editorial'/it OR 'note'/it OR 'animal experiment'/de OR 'animal model'/de OR 'animal tissue'/de OR 'human cell'/de OR 'nonhuman'/de)

Scopus

TITLE-ABS-KEY ("mitral valve insufficiency" OR "mitral regurgitation") OR TITLE-ABS ("mitral insufficiency" OR "mitral regurgitation" OR "mitral valve regurgitation" OR "mitral disease" OR "mitral valve disease" OR "mitral incompetence" OR "mitral valve incompetence") AND TITLE-ABS-KEY ("prevalence" OR "incidence" OR "epidemiology") OR TITLE-ABS ("survey" OR "population-based") AND (EXCLUDE (DOCTYPE , "no") OR EXCLUDE (DOCTYPE , "le") OR EXCLUDE (DOCTYPE , "ed") OR EXCLUDE (DOCTYPE , "cp"))

Appendix S2. Temporal evolution of the definition and assessment methods of moderate-to-severe MR.

Moderate-to-severe mitral regurgitation (MR) has seen substantial advancements in its definition and evaluation over the last few decades, driven by improvements in technology and updates to clinical guidelines. Since the early 1990s, methods for identifying and classifying MR severity have become increasingly refined.

In the early 1990s, MR assessment primarily relied on two-dimensional echocardiography and color Doppler imaging. Evaluations were predominantly qualitative, focusing on visual assessments of the regurgitant jet occupying the left atrium. A jet covering approximately 20-40% of the left atrial area was generally classified as moderate MR, while larger extents suggested severe MR. Quantitative parameters, such as regurgitant volume and effective regurgitant orifice area (EROA), were rarely used, and estimates of regurgitant fraction were often either rough or completely absent.

During the 2000s, the incorporation of detailed and quantitative metrics into clinical practice became more common. Regurgitant volume and fraction were measured with greater consistency, with moderate MR characterized by a regurgitant volume between 30-60 mL and a regurgitant fraction of 30-50%. The vena contracta was introduced as a valuable parameter, with widths exceeding 0.3 cm indicating significant regurgitation, although standardized thresholds had yet to be established. EROA also began to gain wider application, with initial thresholds for moderate MR defined as 0.2-0.39 cm^2.

From 2010 onwards, the advent of advanced imaging technologies such as three-dimensional echocardiography (3D) and cardiac magnetic resonance imaging (MRI) allowed for more precise quantification of MR severity. Guidelines established clearer criteria for severe MR, including a regurgitant volume >60 mL, a regurgitant fraction >50%, and an EROA >0.4 cm^2. For moderate MR, the recognized thresholds included a regurgitant volume of 30-60 mL, a regurgitant fraction of 30-50%, and an EROA of 0.2-0.39 cm^2. The vena contracta measurement was also more precisely defined, with widths ≥0.7 cm considered indicative of severe MR.

Supporting parameters such as elevated pulmonary systolic pressure and evidence of left ventricular remodeling have become increasingly important in comprehensive severity assessments, particularly in more recent studies.

References

1. Zoghbi WA, et al. Recommendations for evaluation of the severity of native valvular regurgitation with two-dimensional and doppler echocardiography. *J Am Soc Echocardiogr.* 2003;16(7):777-802.
2. Lancellotti P, et al. Recommendations for the assessment of valvular regurgitation. Part 2: Mitral and tricuspid regurgitation (native and prosthetic valves). *Eur Heart J Cardiovasc Imaging.* 2013;14(7):612-621.
3. Zoghbi WA, et al. Recommendations for noninvasive evaluation of native valvular regurgitation: a report from the American Society of Echocardiography. *J Am Soc Echocardiogr.* 2017;30(4):303-371.
4. Vahanian A, et al. 2021 ESC/EACTS Guidelines for the management of valvular heart disease. *Eur Heart J.* 2022;43(7):561-632.
5. Lancellotti P, et al. Recommendations for the assessment of valvular regurgitation. Part 1: Aortic and pulmonary regurgitation (native and prosthetic valves). *Eur Heart J Cardiovasc Imaging.* 2013;14(7):611-644.
6. Nagueh SF, et al. Recommendations for the evaluation of left ventricular diastolic function by echocardiography. *J Am Soc Echocardiogr.* 2016;29(4):277-314.

Supplementary Table S1. Methods and criteria used for identifying mitral regurgitation.

Study	Method/criteria for identifying mitral regurgitation	Echocardiographic technique
Berger *et al.,* 1989	Mitral regurgitation diagnosed by systolic velocity signal initiating with mitral closure and occupying ~50% of systole duration	Pulsed and continuous wave Doppler echocardiography
Klein *et al.,* 1990	Mitral regurgitation identified using color flow with 2D Doppler echocardiography	Color Doppler echocardiography
Singh *et al.,* 1999	Moderate mitral regurgitation defined as regurgitant jet area to left atrial area ratio between 20%-40%, severe if >41%	Color Doppler echocardiography
Devereux *et al.,* 2001	Graded on a 4-point system: mild (1+), moderate (2+), moderately severe (3+), severe (4+) based on jet distance from orifice	2D and color Doppler echocardiography
Nkomo *et al.,* 2006	Graded by color Doppler criteria: regurgitant jet occupies ≥1/3 atrial area in any view	Color Doppler echocardiography
Fox *et al.,* 2007	Severity graded by regurgitant jet to atrial area ratio: none, trace (≤5%), mild (>5-20%), moderate (>20-40%), severe (>40%)	2D, color, and spectral Doppler echocardiography
Sandrock *et al.,* 2008	Mitral regurgitation detected using color Doppler	Color Doppler echocardiography
van Bemmel *et al.,* 2010	Severity graded on a qualitative scale (mild, moderate, severe) per ACC/AHA guidelines	Color Doppler echocardiography
Güvenç *et al.,* 2012	Mitral regurgitation assessed from parasternal/apical views, graded as mild or >mild per ASE criteria	2D echocardiography
Vaes *et al.,* 2012	Mitral regurgitation evaluated using ASE's semi-quantitative and quantitative color Doppler methods	Color Doppler echocardiography
Yousaf *et al.,* 2012	Grading based on guidelines from the American and BSE	M-mode, 2D, and Doppler echocardiography
Turker *et al.,* 2015	Mitral regurgitation diagnosed according to ASE recommendations	Color Doppler echocardiography
d'Arcy *et al.,* 2016	Mitral regurgitation defined by valve anatomy, physiology, severity per BSE criteria and international guidelines	Echocardiography
Delling *et al.,* 2016	Mitral regurgitation quantified by color Doppler jet height (>5 mm for moderate)	2D transthoracic and color Doppler echocardiography in parasternal, apical, and subcostal views
Rahimi *et al.,* 2017	Diagnoses primarily based on ICD-10 codes with prior validation for moderate to severe cases showing a positive predictive value >85%	Most cases were confirmed by echocardiography.
Rubin *et al.,* 2019	Mitral regurgitation graded by color Doppler: jet area ≥33% atrial area in line with guideline recommendations	Color Doppler echocardiography
Kim *et al.,* 2019	Standard 2D transthoracic and Doppler echocardiography per ASE/European guidelines	2D transthoracic and Doppler echocardiography
Nazarzadeh *et al.,* 2019	Mitral regurgitation identification through ICD-10 code I34.0 or UK Biobank self-report code 1585	Most cases were confirmed by echocardiography.
Scheel *et al.,* 2019	Mitral regurgitation detected from 2D and color Doppler images in parasternal/apical views, stored in DICOM format	2D and color Doppler echocardiography
He *et al.,* 2021	Mitral regurgitation graded qualitatively with color Doppler; severe defined as grade ≥3/4	Doppler color flow imaging

Abbreviations: ACC, American College of Cardiology; AHA, American Heart Association; ASE, American Society of Ecocardiography; BSE, British Society of Echocardiography; DICOM, Digital Imaging and Communications in Medicine.

Supplementary Table S2. Quality assessment of the included studies.

**Study**	Was the sample frame appropriate to address the target population?	Were study participants sampled in an appropriate way?	Were study subjects and setting described in detail?	Was the data analysis conducted with coverage of the identified sample?	Were valid methods used for the idenfication of the condition?	Was the condition measured in a standard, reliable way for all parcipants?	Was the response rate adequate, and if not, was the low response rate managed appropriately?
Berger *et al.*, 1989	YES	UNCLEAR	YES	YES	YES	YES	YES
Klein *et al.*, 1990	YES	UNCLEAR	YES	YES	YES	YES	YES
Singh *et al.*, 1999	YES	YES	YES	YES	YES	YES	YES
Devereux *et al.*, 2001	YES	YES	YES	YES	YES	YES	YES
Nkomo *et al.*, 2006	YES	YES	YES	YES	YES	YES	YES
Fox *et al.*, 2007	YES	YES	YES	YES	YES	YES	YES
Sandrock *et al.*, 2008	YES	YES	YES	YES	YES	YES	YES
van Bemmel *et al.*, 2010	YES	YES	YES	YES	YES	YES	YES
Güvenç *et al.*, 2012	UNCLEAR	YES	YES	YES	YES	UNCLEAR	YES
Vaes *et al.*, 2012	YES	YES	YES	YES	YES	YES	YES
Yousaf *et al.*, 2012	YES	YES	YES	NO	YES	YES	YES
Turker *et al.*, 2015	YES	YES	YES	YES	YES	YES	YES
d'Arcy *et al.*, 2016	YES	YES	YES	YES	YES	YES	YES
Delling *et al.*, 2016	YES	YES	YES	YES	YES	YES	YES
Rahimi *et al.*, 2017	YES	YES	YES	YES	YES	NO	N/A
Rubin *et al.*, 2019	YES	YES	YES	YES	YES	YES	YES
Kim *et al.*, 2019	UNCLEAR	YES	YES	YES	YES	YES	YES
Nazarzadeh *et al.*, 2019	YES	YES	YES	YES	UNCLEAR	UNCLEAR	N/A
Scheel *et al.*, 2019	YES	YES	YES	YES	YES	UNCLEAR	YES
He *et al.*, 2021	YES	YES	YES	YES	YES	YES	YES

Abbreviation: N/A, Not Applicable.

Supplementary Table S3. Sensitivity multivariable mixed-effects meta-regression analysis reporting the independent association of mean age, proportion of males, study year, and continent, with prevalence of moderate-to-severe mitral regurgitation.

Moderator	N. of estimates	β coefficient	(95% CI)	*P*-value
Age	50	+0.0048	(+0.0032, +0.0063)	<0.0001
Proportion of males	50	+0.0082	(-0.0605, +0.0768)	0.8156
Study year	50	-0.0028	(-0.0065, +0.0009)	0.1357
Continent	50	...	...	...
Africa	...	Reference	...	...
Asia	...	-0.1381	(-0.3212, +0.0450)	0.1394
Europe	...	-0.1065	(-0.2848, +0.0717)	0.2415
North America	...	-0.1557	(-0.3405, +0.0291)	0.0987

Abbreviation: CI, Confidence Interval.
Footnote: Beta-coefficients are presented on the Freeman-Tukey scale, which limits direct interpretability.

Supplementary Table S4. Sensitivity multivariable mixed-effects meta-regression analysis reporting the independent association of mean age, proportion of males, study year, and predominant ethnicity, with prevalence of moderate-to-severe mitral regurgitation.

**Variable**	**N. of estimates**	**β coefficient**	**(95% CI)**	***P*-value**
Age	50	+0.0043	(+0.0028, +0.0059)	<0.0001
Proportion of males	50	+0.0044	(-0.0683, +0.0771)	0.9055
Study year	50	-0.0008	(-0.0045, +0.0028)	0.6556
Predominant ethnicity	50	..	..	..
American Indian	..	Reference		
Asian	..	+0.0111	(-0.1946, +0.2168)	0.9158
Black	..	-0.0092	(-0.1961, +0.1778)	0.9235
Hispanic	..	+0.0234	(-0.1687, +0.2154)	0.8117
White	..	+0.0308	(-0.1369, +0.1985)	0.7189

Abbreviation: CI, Confidence Interval.
Footnote: Beta-coefficients are presented on the Freeman-Tukey scale, which limits direct interpretability.

Supplementary Figure S1. Funnel plot of the prevalence of moderate-to-severe mitral regurgitation in adults (N=17).

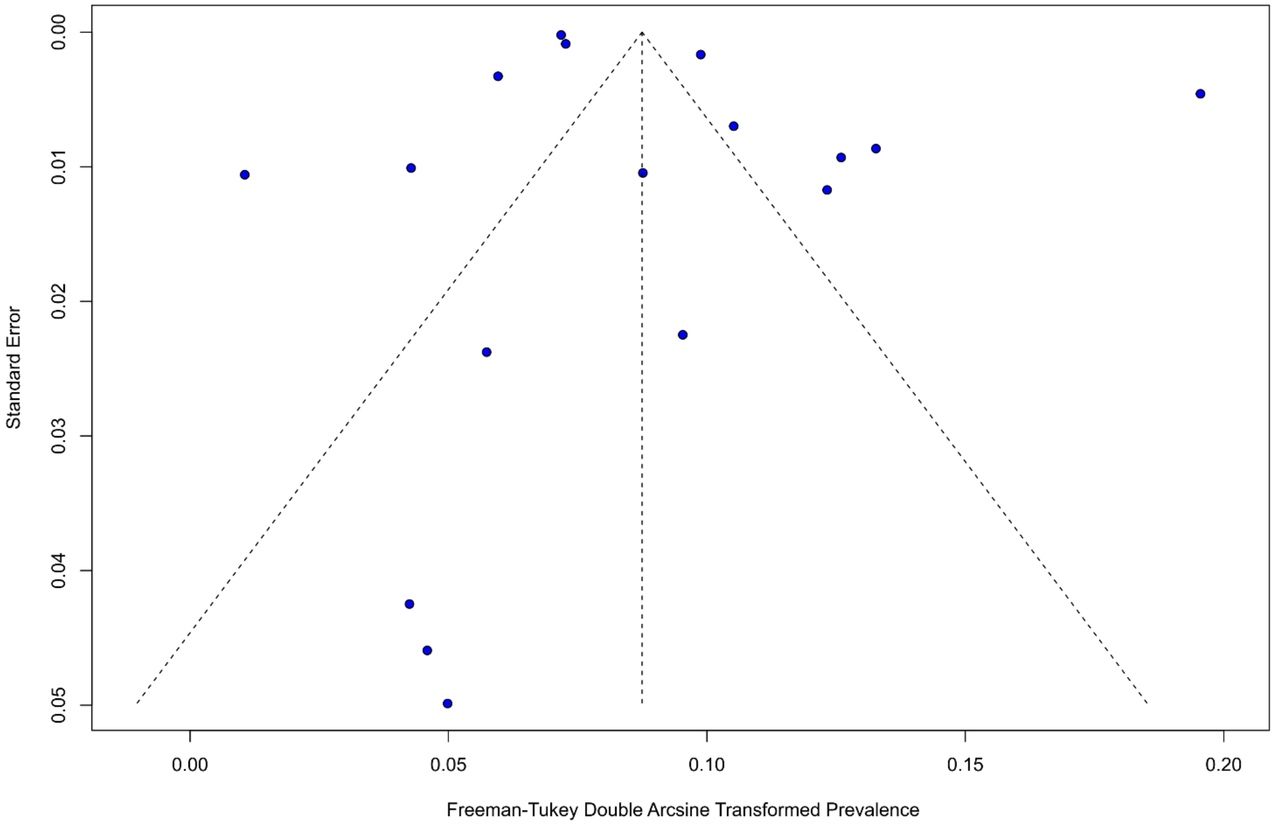

Supplementary Figure S2. Subgroup analysis of the prevalence of moderate-to-severe mitral regurgitation in adults, by predominant ethnicity of participants (N=17).

Study	Events	Participants	Prevalence [95% CI]	weight
**White**				
Berger et al., 1989	0	100	0.0000 [0.0000; 0.0362]	3.0%
Klein et al., 1990	0	118	0.0000 [0.0000; 0.0308]	3.3%
Singh et al., 1999	45	2881	0.0156 [0.0114; 0.0208]	6.5%
Nkomo et al., 2006 (CARDIA, ARIC, CHS)	449	11911	0.0377 [0.0342; 0.0412]	6.8%
Nkomo et al., 2006 (Olmsted county)	874	89853	0.0097 [0.0091; 0.0104]	6.8%
Sandrock et al., 2008	56	5124	0.0109 [0.0084; 0.0143]	6.7%
Güvenç et al., 2012 (Instanbul)	1	442	0.0023 [0.0006; 0.0134]	5.3%
Güvenç et al., 2012 (Kars)	4	494	0.0081 [0.0030; 0.0215]	5.4%
Turker et al., 2015	0	2228	0.0000 [0.0000; 0.0009]	6.5%
Delling et al., 2016	0	138	0.0000 [0.0009; 0.0287]	3.5%
Rahimi et al., 2017	28582	5553984	0.0051 [0.0049; 0.0050]	6.8%
Nazarzadeh et al., 2019	1736	329237	0.0053 [0.0021; 0.0025]	6.8%
**Random effects model**		**5996510**	**0.0062 [0.0020; 0.0121]**	**67.3%**
Heterogeneity: $I^2 = 99\%$, $\tau^2 = 0.0023$, $p < 0.01$				
**American Indian**				
Devereaux et al., 2001	58	3340	0.0174 [0.0134; 0.0225]	6.6%
**Black**				
Fox et al., 2007	17	2284	0.0074 [0.0043; 0.0118]	6.5%
Scheel et al., 2019	4	2453	0.0016 [0.0006; 0.0043]	6.5%
**Random effects model**		**4737**	**0.0040 [0.0003; 0.0116]**	**12.9%**
Heterogeneity: $I^2 = 89\%$, $\tau^2 = 0.0009$, $p < 0.01$				
**Hispanic**				
Rubin et al., 2019	27	1818	0.0149 [0.0101; 0.0218]	6.4%
**Asian**				
Kim et al., 2019	82	23254	0.0035 [0.0026; 0.0042]	6.8%
**Random effects model**		**6029659**	**0.0067 [0.0033; 0.0111]**	**100.0%**
Heterogeneity: $I^2 = 99\%$, $\tau^2 = 0.0019$, $p < 0.01$				
Test for subgroup differences: $\chi^2_4 = 83.59$, df = 4 ($p < 0.01$)				

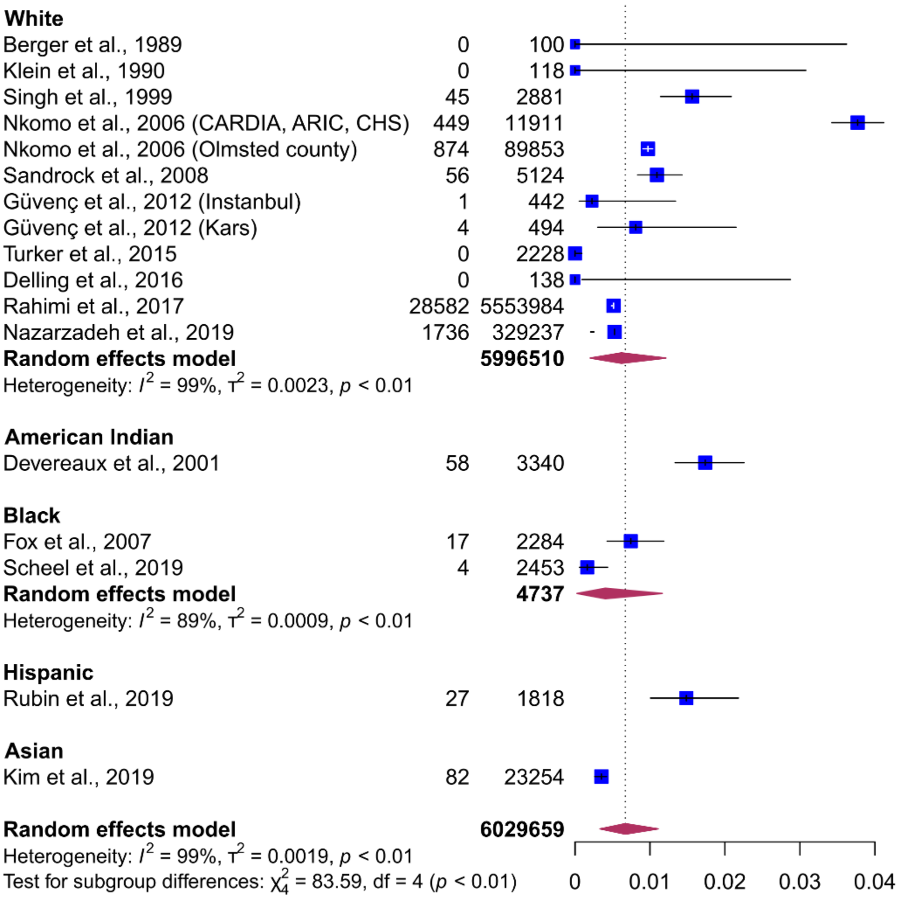

Supplementary Figure S3. Bubble plot from univariable meta-regression analysis, illustrating the association of mean age with the prevalence of moderate-to-severe mitral regurgitation.

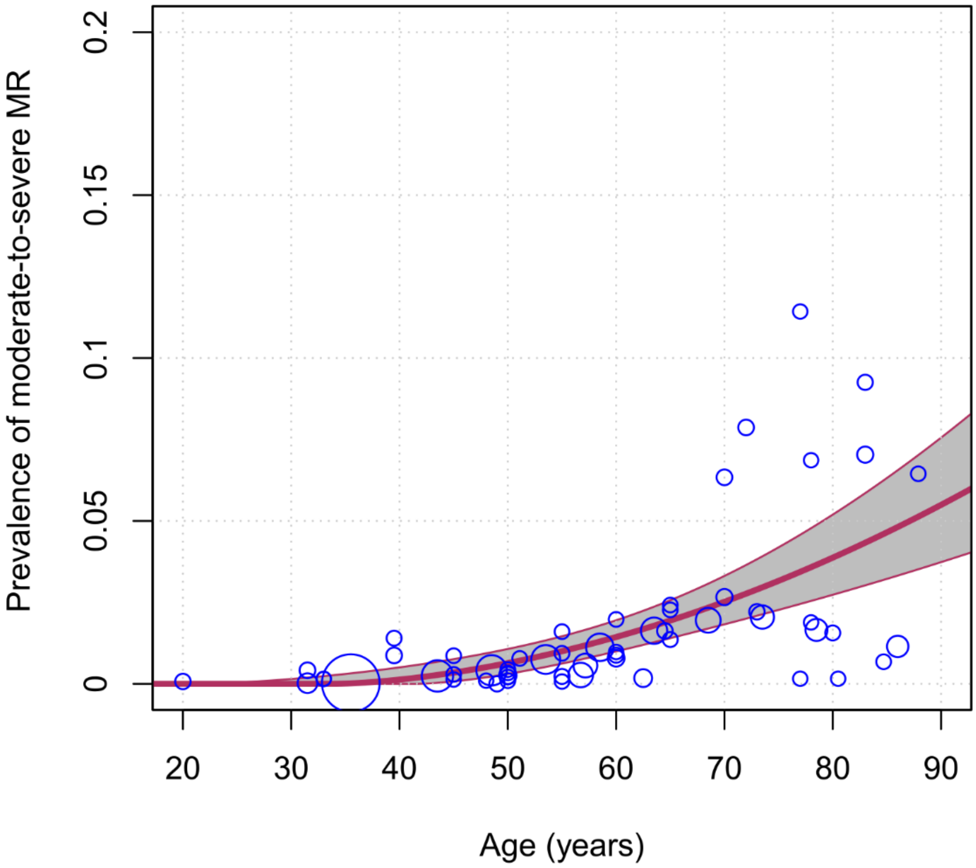

Footnotes: The study-specific effect size is displayed on the y-axis and the moderator of interest on the x-axis. The size of the markers is proportional to the size of each study. The graphs are accompanied by the (marginal) multivariable meta-regression lines in red and the respective 95% confidence intervals in grey. The bubble representing the study by van Bemmel et al. (2010) is omitted to enhance visual clarity, though its data were fully incorporated in the analysis.

Supplementary Figure S4. Bubble plot from univariable meta-regression analysis, illustrating the association of proportion of males with the prevalence of moderate-to-severe mitral regurgitation.

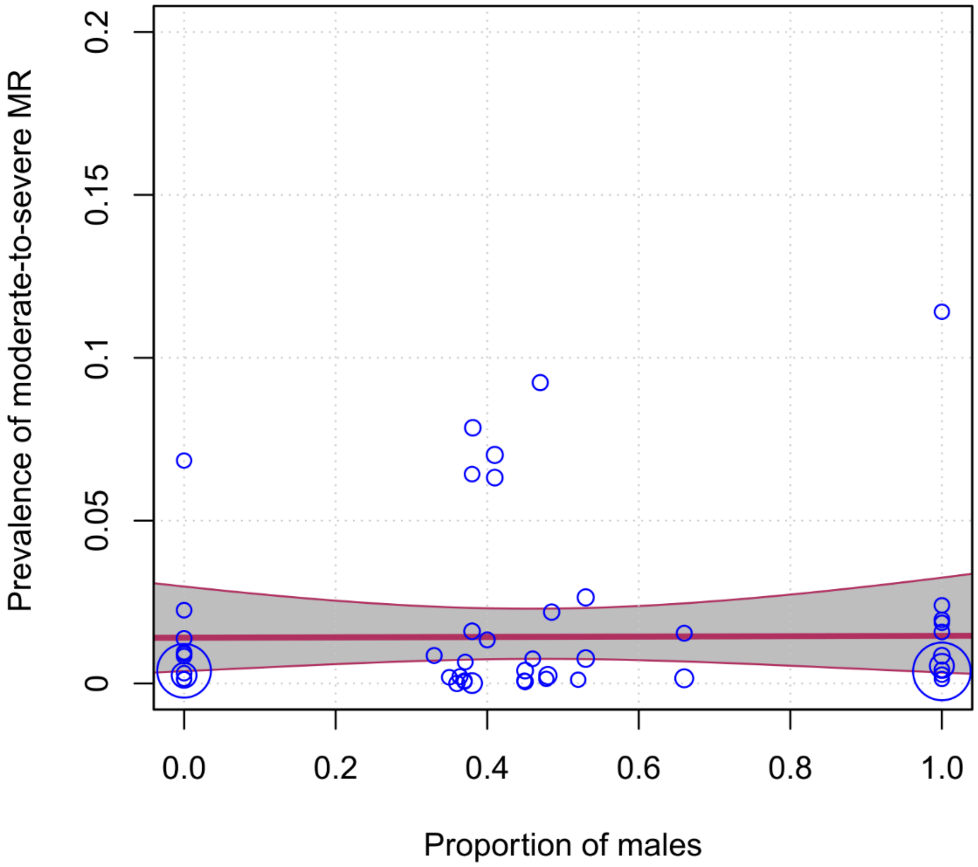

Footnotes: The study-specific effect size is displayed on the y-axis and the moderator of interest on the x-axis. The size of the markers is proportional to the size of each study. The graphs are accompanied by the (marginal) multivariable meta-regression lines in red and the respective 95% confidence intervals in grey. The bubble representing the study by van Bemmel et al. (2010) is omitted to enhance visual clarity, though its data were fully incorporated in the analysis.

Supplementary Figure S5. Bubble plot from univariable meta-regression analysis, illustrating the association of study year with the prevalence of moderate-to-severe mitral regurgitation.

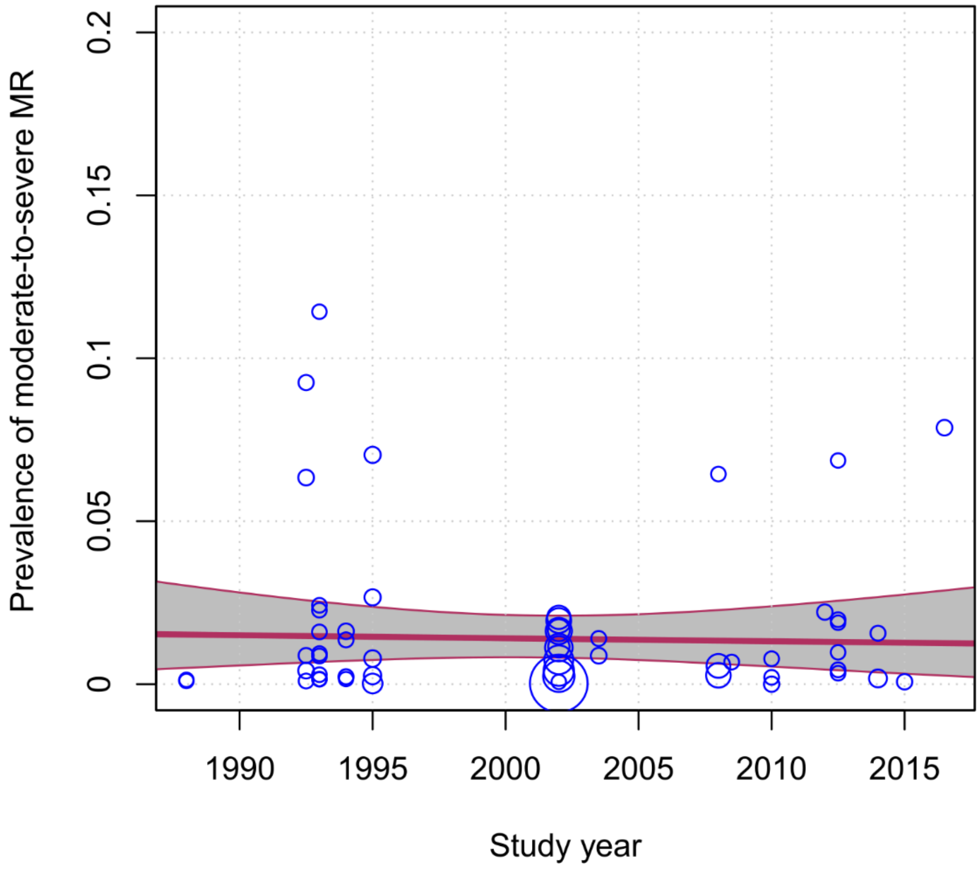

Footnotes: The study-specific effect size is displayed on the y-axis and the moderator of interest on the x-axis. The size of the markers is proportional to the size of each study. The graphs are accompanied by the (marginal) multivariable meta-regression lines in red and the respective 95% confidence intervals in grey. The bubble representing the study by van Bemmel et al. (2010) is omitted to enhance visual clarity, though its data were fully incorporated in the analysis.
